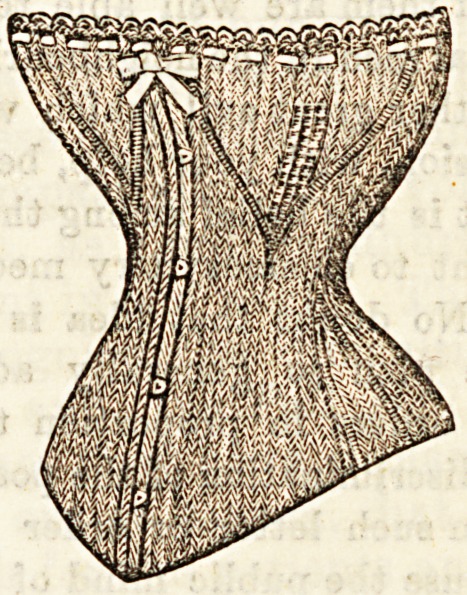# New Drugs, Appliances, and Things Medical

**Published:** 1891-12-26

**Authors:** 


### NEW DRUGS, APPLIANCES, AND THINGS MEDICAL.

[All preparations, appliances, novelties, etc., of which a notice is desired, should be sent for The Editor, to care of The Manager, 140, Strand, London, W.C.]

### VACCINE CASE.

JOHN WEISS AND SON, 287, OXFORD STREET, LONDON.

The above firm have sent us for notice an extremely well-made and ingenious case for vaccine tubes. It is strongly made, lined with velvet, and covered with leather. On pulling out the "drawer" and lifting up its protecting flap, twelve divisions are seen. Thus the tubes taken in every month can be kept separate, and any note concerning them can be slipped into the appropriate division. We think the firm have succeeded in supplying a want.

### CELLULAR CLOTH CLOTHING.

We have already recommended this special fabric for underclothing, to our readers, and at this season when we are all liable to suffer from the effects of cold, we venture again to bring a useful preventive to their notice. The cellular cloth is light, remarkably warm, and is manufactured on scientific principles, which admit of a free circulation of air. It is, morever, fashioned into shapely garments of all descriptions at a moderate price.

### ANTI-RHEUMATIC TOWELS.

To those who have not yet procured one of the Bellhouse Electric Anti-Rheumatic Towels we can confidently say, do so. As an additional benefit after the morning tub it is excellent. Apart from the anti-rheumatic qualities which it claims, it produces such an agreeable warmth to the body, that once used after the bath in winter it is not likely to be thrown aside. The same company manufactures underclothing of a like material, which resembles a mixture of silk and cotton, and is said to ward off cold more effectually than flannel. The towels vary from 2s. 6d. to 6s. 6d., according to size and quality, and are to be procured from the company, 4, Marsden Square, Manchester.

### KNITTED CORSETS.

The Sanitary Knitted Corset Company offer the public a most seasonable and useful article of women's clothing in the corset as shown in illustration. We can imagine no more suitable gift where it is desired to make a useful present to an aged or delicate friend. The woollen corsets render extra flannel garments unnecessary, and admit of ventilation at the same time. The price, ranging from 6s. 6d. to 9s. 6d., place the corsets within the reach of most people, and they can be had cheaper in other knitted materials, where warmth is not a *sine quâ non*. The same company supplies us with patterns of most excellent elastic woollen materials for underclothing, and for these we recommend our friends of the fair sex to write to 44, Mansfield Road, Nottingham.

### LOBECK'S PURE SOLUBLE COCOA.

LOBECK AND CO., DRESDEN.

Samples of the above have been forwarded to us for examination and notice. It is stated to be manufactured by a steam-pressure process, and to contain no soda, potash, or other chemicals as used in the ordinary Dutch process. Our examination bears this out. It is a clean, well-made, excellent-flavoured cocoa powder, answering to all the usual tests for a pure cocoa. It forms, when mixed in the ordinary way, a first-class, highly-nutritious beverage for breakfast or other meals. We note that Messrs. Lobeck have taken many medals and highest awards in the last twelve years at the various home and colonial exhibitions.

### CUCA COCOA.

ROOT AND CO. (LIMITED), 38, GREAT RUSSELL STREET, W.C.

This is a well-made cocoa, which is stated to contain "the extractive value of a certain weight of the dried leaves of the 'Erythroyton cocoa' plant, to a given weight of pure cocoa." Accordingly, the samples submitted to us were examined for the characteristics of pure cocoa, and for the active principle of the cuca leaf—cocaine. The result proved that the above statement is correct. The cocoa is a first-class article, and it contains cocaine. It is claimed for the latter that it is a gradual restorative and sustainer, or tonic. It seems likely, therefore, that the ingenious combination of cocoa with cuca leaf will form a product which is at once nutritious, tonic, and stimulating, without the disadvantages of alcohol. We imagine it will suit those who have objections to wine, and so cannot or will not take even medicinal wines, such as cocoa wine, and also the large class of athletes, and those engaged in laborious and exhausting occupations, placing before them their stimulant in a pleasing and palatable form.

## Figures and Tables

**Figure f1:**
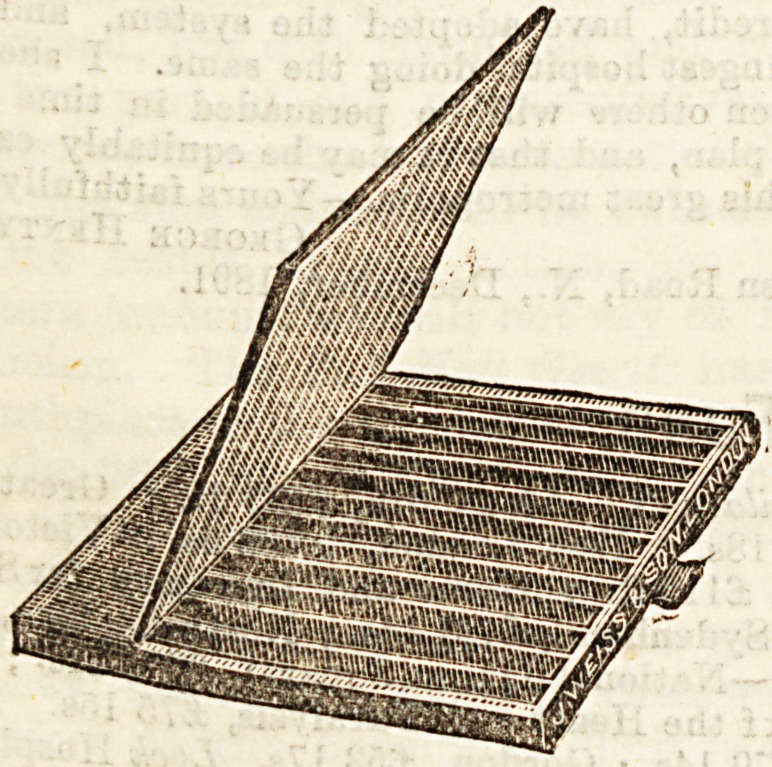


**Figure f2:**